# Vitamin D and Hashimoto’s Thyroiditis: Observations from CROHT Biobank

**DOI:** 10.3390/nu13082793

**Published:** 2021-08-15

**Authors:** Maja Cvek, Dean Kaličanin, Ana Barić, Marko Vuletić, Ivana Gunjača, Vesela Torlak Lovrić, Veselin Škrabić, Ante Punda, Vesna Boraska Perica

**Affiliations:** 1 Department of Nuclear Medicine, University Hospital of Split, 21000 Split, Croatia; maja.cvek.st@gmail.com (M.C.); ana.baaric@gmail.com (A.B.); mavuletic@gmail.com (M.V.); veselakbsplit@yahoo.com (V.T.L.); ante.punda@gmail.com (A.P.); 2 Department of Medical Biology, School of Medicine, University of Split, 21000 Split, Croatia; dkalican@mefst.hr (D.K.); igunjaca@mefst.hr (I.G.); 3 Department of Pediatrics, University Hospital of Split, 21000 Split, Croatia; vskrabic@kbsplit.hr

**Keywords:** vitamin D, Hashimoto’s thyroiditis, thyroid autoimmunity, thyroid stimulating hormone (TSH), thyrotropin

## Abstract

The aims of this study were to evaluate: (1) associations of vitamin D with the presence/severity of Hashimoto’s thyroiditis (HT) and (2) correlations of vitamin D with thyroid-related phenotypes. Total 25(OH)D (vitamin D in the text) was measured from stored serum samples of 461 HT patients and 176 controls from a Croatian Biobank of HT patients (CROHT). (1) Vitamin D levels, and proportions of vitamin D deficiency, were compared between HT cases and controls. HT patients were additionally divided into two groups (MILD and OVERT) to take into account HT severity. (2) Correlations between vitamin D and 10 clinical phenotypes in all HT patients and two subgroups of HT patients were tested using the Spearman correlation test. Our analyses were adjusted for age, gender, BMI, smoking status and seasonality of blood sampling. (1) No significant differences in vitamin D levels, or proportions of vitamin D deficiency, were detected between HT patients of all disease stages and controls. However, a nominally significant difference in vitamin D levels between MILD and OVERT subgroups (OR = 1.038, *p* = 0.023) was observed. Proportions of individuals with vitamin D deficiency during winter–spring were high: all HT cases (64.69%), MILD (60.64%), OVERT (68.7%), controls (60.79%). (2) A nominally significant negative correlation between vitamin D and TSH in all HT patients (r = −0.113, *p* = 0.029) and a positive correlation between vitamin D and systolic blood pressure in OVERT HT patients (r = 0.205, *p* = 0.025) were identified. Our study indicates that there is no association between vitamin D and HT; however, there may be a subtle decrease in vitamin D levels associated with overt hypothyroidism.

## 1. Introduction

Vitamin D, traditionally known as a fat-soluble vitamin, is a confirmed steroid hormone with its main role in calcium and phosphorus homeostasis and the regulation of bone metabolism. The term “vitamin D” stands for two compounds: cholecalciferol (vitamin D3) and ergocalciferol (vitamin D2) [1]. Vitamin D3 is synthesized in the skin (about 80%) by sunlight exposure (UVB radiation) while vitamin D2 is produced by plants, fungi and yeast (about 20%) [1,2]. Humans get vitamin D from three different sources: skin production, diet and supplementation.

Both forms of vitamin D are hydroxylated to 25-hydroxyvitamin D, 25(OH)D or calcidiol, which is the major circulating form of vitamin D with a half-life of 2–3 weeks [3]. 25(OH)D is measured and used as an indicator of the vitamin D status in an organism. The first hydroxylation takes place in the liver, while the second one occurs in the kidney by the action of 1-α-hydroxylase, CYP27B1, where calcidiol is hydroxylated to calcitriol (1,25(OH)2D), the biologically active form of vitamin D [4]. 1,25(OH)2D binds to the nuclear vitamin D receptor (VDR) in target tissues [5] thus regulating the expression of more than 200 genes (3–5% of the human genome) [6]. VDR and the enzyme CYP27B1 are not only found in the small intestine, skeletal and renal cells, as was thought earlier, but in almost all body cells, including thyroid and immune cells [7].

Sufficient levels of vitamin D are important for human health. The role of vitamin D is shown in a variety of endocrine and autoimmune diseases (type 1 diabetes, type 2 diabetes, polycystic ovary syndrome, adrenal diseases, multiple sclerosis, systemic lupus erythematosus, rheumatoid arthritis, AITD), cancers, inflammatory responses, infectious and cardiovascular diseases [8,9]. According to the Endocrine Society Guidelines, a sufficiency of vitamin D is considered when serum 25(OH)D is equal to or greater than 30 ng/mL and up to 100 ng/mL (≥75 nmol/L up to 250 nmol/L), insufficiency is considered when vitamin D levels are between 20 and 29.9 ng/mL, while vitamin D deficiency is considered when serum 25(OH)D is below 20 ng/mL [10]. The worldwide prevalence of vitamin D deficiency is high [11,12] and for Europeans it is estimated to be about 40% [13].

Hashimoto’s thyroiditis (HT), chronic lymphocytic or autoimmune thyroiditis, is one of the thyroid diseases with a prevalence of about 10–12% and an increasing incidence, and is considered to be the most frequent thyroid disorder worldwide [14,15]. Age, gender and race are known HT modifiers, thus white women from 45 to 55 years of age, are four to ten times more affected by HT than males [16]. Main HT characteristics are lymphocyte infiltration into the thyroid gland, causing progressive tissue destruction and the production of antithyroid antibodies, thyroid peroxidase antibodies (TPOAb) and thyroglobulin antibodies (TgAb) [17]. Thyroid autoantibodies are, together with thyroid ultrasound, characteristic features and useful markers for HT diagnosis [18]. The clinical manifestation of disease ranges from mild, characterized by the presence of either one or both types of thyroid antibodies in euthyroid patients, to the gradual development to subclinical and overt hypothyroidism, with or without goiter [18]. HT is caused by the combined action of genetic (about 70%), environmental (20–30%) and existential factors [19]. The most known environmental risk factor for HT is iodine excess, while other suggested ones are, infections (viral and bacterial), medications (e.g., interferon α, amiodarone), and chemicals (e.g., polyaromatic hydrocarbons or polyhalogenated biphenyls) [20,21,22,23]. In the focus of scientific investigations, as potential environmental factors, there are vitamin D, selenium and gluten [24,25,26], as well as dietary habits. More work is needed to identify the impact of environmental factors on disease appearance and/or its progression.

The (patho)physiological mechanism of vitamin D involvement in HT development has not been clearly explained yet. The first report about the relationship between vitamin D and thyroid autoimmunity was published in 2009 [27] when Goswami et al. showed an inverse correlation between 25(OH)D and TPOAb in 642 adults (teachers, students, staff). Since then, a large number of studies tried to clarify the association of vitamin D with HT by analyzing differences in vitamin D levels between HT cases and control participants, but without reaching consistent results. The most recent study found lower vitamin D levels in 373 HT cases in comparison to 4889 control participants who had vitamin D measured at the same time; however, after performing multivariate logistic regression the study concluded that vitamin D was not associated with HT [28]. Another recent systematic review, meta-analysis and meta-regression of observational studies [29] reported a significant association between vitamin D status and HT, suggesting that HT patients have lower serum 25(OH)D concentrations than those without HT. This meta-analysis investigated and summarized data across twenty-five studies consisting of 2695 cases and 2263 control individuals. However, the majority of studies were of a small sample size, and only four of them had more than 200 subjects in the HT group. More importantly, many studies that have not found an association between vitamin D and HT were not incorporated in this meta-analysis [30,31,32,33]. The weakness of these studies is the limited number of participants.

Given the inconclusive results on the relationship between vitamin D and HT, we aimed to perform a comprehensive set of analyses, in a large group of clinically diagnosed HT patients and control individuals from the Croatian Biobank of HT patients (CROHT biobank) [34,35] to give more conclusive results on this topic. A recent systematic review of vitamin D concentrations in relation to HT [29] pointed to several moderators that contribute to patient heterogeneity. Besides known factors that can modify vitamin D levels, such as age [36], sex [37], BMI [38] and seasonality [39,40,41], there are many others that are usually overlooked when studying vitamin D levels, such as smoking, severity of illness, outdoor activity, education, occupation or income [29]. In our study, we used all available information from the CROHT biobank to extrapolate as many of these potential “vitamin D modifying factors” as possible. We therefore used adjustments for age, sex, BMI, smoking and seasonality to minimize the impact of these factors on patient vitamin D levels. We also stratified our HT cases according to HT severity and performed sub-analyses in these groups to take into account the severity of illness. Taken altogether, our study has several aims: the first one is to determine if vitamin D levels differ between HT cases (and HT cases stratified by severity) and controls, the second one is to evaluate the proportions of vitamin D deficiency in study participants, and the final one is to extensively evaluate correlations between vitamin D levels and thyroid-specific phenotypes in HT patients and the subgroups of patients according to illness severity.

## 2. Materials and Methods

Subjects: This is a retrospective observational study in which we used stored serum samples for vitamin D measurements, and phenotype information from HT patients and control participants from the CROHT biobank [34,35]. Total 25(OH)D (vitamin D in the text) was measured in 461 HT patients (92.41% females) and 176 control participants (93.75% females) by the LIAISON 25(OH) Vitamin D Total chemiluminescence immunoassay (DiaSorin, Saluggia, Italy). All study subjects from the CROHT biobank were over the age of 18 and during the recruitment period were examined by clinical specialists in nuclear medicine at the Outpatient clinic for thyroid disorders in the Clinical Department of Nuclear Medicine at the University Hospital of Split. HT patients were recruited from 2015 to 2017, covering all seasons, whereas control participants were recruited from December 2018 till June 2019, covering winter and spring. All subjects that used medications and dietary supplements that may impact vitamin D levels (corticosteroids, anticonvulsants, vitamin D supplementation or daily use of calcium supplementation), were excluded from this study.

Diagnosis of HT was made following the ETA recommendations and guidelines for the Management of Subclinical Hypothyroidism [42]. The inclusion criteria for HT patients were: (1) echographic pattern of diffuse thyroid disease; (2) increased thyroid-stimulating hormone (TSH) and/or decreased thyroid hormones—triiodothyronine (T3), thyroxine (T4) or free thyroxine (fT4) and/or increased thyroid autoantibodies (TPOAb and TgAb). The absence of HT in control participants was also established on the basis of clinical examination, following these criteria: (1) echographic pattern of homogenous, normoechogenic (isoechogenic) thyroid parenchyma, with an absence of any diffuse or focal lesions; (2) TSH, T3, T4, fT4, TPOAb and TgAb within reference ranges: TSH (0.3–3.6 mIU/L), T3 (1.3–3.6 nmol/L), T4 (57.3–161 nmol/L), fT4 (10.3–22.8 pmol/L), TPOAb (1–16 IU/mL), TgAb (5–100 IU/mL).

Multiple phenotypes were directly measured in study participants or collected using a questionnaires during their enrolment in the CROHT biobank: thyroid related traits (TSH, T3, T4, fT4, TPOAb, TgAb, thyroid gland ultrasound, levothyroxine (LT4) intake, presence/absence of the 16 most common symptoms of hypothyroidism), information on personal anamnesis, comorbidities, main anthropometric and cardiovascular features, information on medical treatments, use of drugs, smoking status, physical activity, dietary habits and many others. During the recruitment stage, blood samples were collected from each participant and used for the measurement of thyroid hormones and antibodies (TSH, T3, T4, fT4, TgAb and TPOAb) using LIAISON chemiluminescence immunoassays (DiaSorin Saluggia, Italy). The remaining serum/plasma was stored in a freezer at −80 °C. The thyroid volume was calculated using dimensions obtained by thyroid gland ultrasound, as a sum of the volumes of both lobes of the thyroid gland. The volume of each lobe was calculated as length × width × depth × 0.479 [43]. The main clinical features of study participants are summarized in Table 1.

All study participants signed an Agreement for participation prior to inclusion in the study. Two Ethics Committees, one from the University of Split School of Medicine (Classification no. 003-08/14-03/0001 and Registry no. 2181-198-03-04-14-0028; Classification no. 003-08/19-03/0003 and Reg. no. 2181-198-03-04-19-0019) and the other from the University Hospital of Split (Classification no. 530-02/13-01/11; Registry no. 2181-147-01/06/J.B.-14-2; Classification no. 500-03/18-01/80 and Reg. no. 2181-147-01/06/M.S.-18-2) approved this research and declared that it was in accordance with the provisions of the Code of Ethics and the Helsinki Declaration.

Statistical analyses: A sample size was calculated prior to performing statistical analyzes (available online: https://www.stat.ubc.ca/~rollin/stats/ssize/ (accessed on 11 August 2021)). In order to achieve a study power of 80% with a type I error rate of 0.05, the number of samples for each group should be at least 121 individuals. To analyze differences in vitamin D levels between 461 HT patients and 175 controls, a logistic regression model was used, where case/control status was used as the dependent variable and vitamin D as an independent variable, along with age, gender, BMI, smoking status and seasonality of blood sampling (a seasonality of sampling was missing for one control, therefore, it was omitted from this analysis). An identical analysis using a subset of 313 HT patients that were recruited during the same time span as control individuals, covering winter and spring, was additionally performed.

In the subsequent analysis, our HT patients were classified into two subgroups depending on HT severity at the time of recruitment. The first group, MILD, incorporated 240 HT patients that were euthyroid (TSH within reference ranges) or in subclinical hypothyroidism (TSH within the range of 3.6–10 mIU/L). The second group, OVERT, contained 219 patients that were in overt hypothyroidism (TSH > 10 mIU/L) or were treated with levothyroxine (LT4) therapy due to previously established overt hypothyroidism (two HT patients were not reallocated in these two groups due to missing data on their therapy status). Differences in vitamin D levels were compared between each of the two subgroups of HT patients with control participants and additionally using a subset of MILD (155) and OVERT (147) patients that matched the controls for the season of the blood draw. Additionally, an in-between comparison of the two subgroups of HT patients using a logistic regression model was performed, adjusting for the same covariates as in the main case-control comparison.

Proportions of vitamin D deficiency (<20 ng/mL) were evaluated in all groups (all HT cases, MILD, OVERT and controls). We were also interested to compare the proportions of individuals with vitamin D deficiency between HT cases (all HT cases, MILD and OVERT) with control participants using the χ2-test. However, for these analyses only HT cases that matched controls for the season of blood draw were selected. Proportions of individuals with vitamin D deficiency were compared between the two subgroups of HT patients, using all samples, because the recruitment of HT cases was uniformly distributed throughout the year.

The next important goal of our study was to examine correlations between vitamin D and clinical phenotypes in all HT patients and the two subgroups of HT patients, using the Spearman correlation test. We tested 10 clinical phenotypes: thyroid hormones (TSH, T3, T4, fT4), thyroid antibodies (TPOAb and TgAb), thyroid volume, number of hypothyroidism symptoms, systolic and diastolic blood pressure. The Bonferroni corrected *p*-value of 0.005 (0.05/10) was used as a significance threshold. Prior to correlation analyses, vitamin D levels were corrected for age, gender, BMI, smoking and seasonality of blood sampling, whereas all clinical phenotypes were corrected for LT4 therapy status (yes/no) in all HT patients and OVERT cases using the linear regression model. All statistical analyses were performed using SPSS (version 20) statistical software.

## 3. Results

No significant differences in median vitamin D levels were detected between: (1) HT patients and control participants; HT cases and controls that matched for the seasonality of blood sampling; (2) MILD cases and controls; MILD cases and controls that matched for the seasonality of blood sampling; (3) OVERT cases and controls; OVERT cases and controls that matched for the seasonality of blood sampling (Table 2). However, a nominally significant difference in vitamin D levels between MILD and OVERT subgroups of HT cases was observed (Table 2).

**Figure 1:**
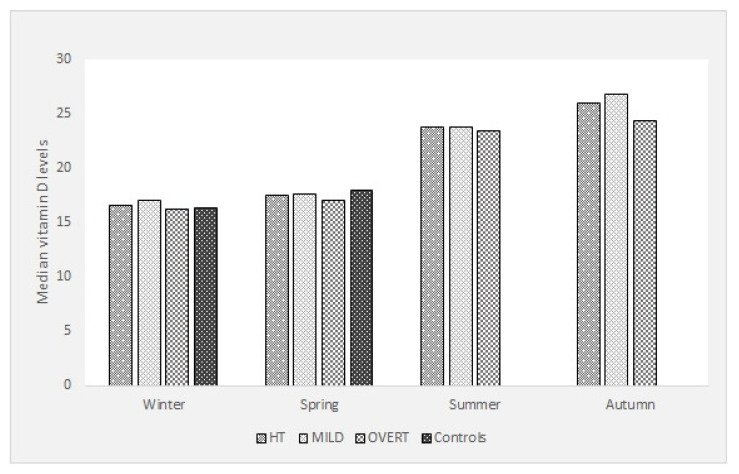
Seasonal distribution of vitamin D levels between groups of participants.

The distribution of vitamin D levels for all HT cases, MILD, OVERT and controls over the four seasons of the year is shown in Figure 1. Vitamin D levels show an expected distribution with the lowest values in winter/spring and highest in summer/autumn. Proportions of individuals with vitamin D deficiency were high in all the examined groups throughout all the year: all HT cases (51.84%), MILD (47.92%), OVERT (56.16%). Proportions of individuals with vitamin D deficiency in the groups of HT cases that matched the controls for the season of blood draw (winter–spring) are, expectedly, higher: all HT cases (64.69%), MILD (60.64%), OVERT (68.7%) and controls (60.79%). Proportions of individuals with vitamin D deficiency in the groups of HT cases for the summer–autumn are lower: all HT cases (18.94%), MILD (12%), OVERT (27.23%). Results of the comparison of proportions of individuals with vitamin D deficiency between HT cases, MILD and OVERT that matched the controls for the season of a blood draw and control participants, and, in-between MILD and OVERT cases (for all the year) are presented in Table 3. There were no significant differences in proportions of vitamin D deficiency between all the investigated groups.

Correlations between vitamin D levels and clinical phenotypes in all HT patients and the two subgroups of HT depending on severity (MILD and OVERT) are shown in Table 4. There were no Bonferroni corrected statistically significant correlations between vitamin D levels and clinical phenotypes, although two nominally significant correlations were identified (*p* < 0.05): (1) a weak negative correlation between vitamin D and TSH in all HT patients (r = −0.113, *p* = 0.029) and (2) a weak positive correlation between vitamin D and systolic blood pressure in OVERT HT patients (r = 0.205, *p* = 0.025).

## 4. Discussion

We performed a comprehensive study on associations between vitamin D levels and HT using a large group of deeply phenotyped HT cases and controls from the CROHT biobank. The main result of our cross-sectional and retrospective study is that we have not detected differences in vitamin D levels nor in proportions of vitamin D deficiency between all HT cases (HT patients of all disease stages) and controls. However, we detected a nominally significant difference in vitamin D levels between the MILD and OVERT subgroups of HT cases.

Our result of no association between vitamin D and HT is not supported by the recent systematic study and meta-analysis across 25 studies that reported a positive association of vitamin D levels and HT [29]. However, the same study concluded that many factors that influence patient heterogeneity, are frequently not included in analyses. The same conclusion was made by D’Aurizio et al. in a review paper from 2015, that pointed out that factors such as heterogeneity of the study population, seasonality of blood withdrawal, analytical variability of vitamin D assays and various definitions for declaring vitamin D deficiency may contribute to inconsistent findings [44]. Moreover, most of the studies that found a positive association between vitamin D levels and HT have had small numbers of participants with limited study designs [44]. For that reason, we performed the largest study to date including 461 clinically diagnosed HT patients and 176 clinically examined HT-free control participants from the same geographical region. The other advantage of our study, in comparison to the majority of other studies performed to date, is that we adjusted our analyses for the most comprehensive set of factors that are potential modifiers of individual vitamin D levels (age, sex, BMI, smoking status and season of blood sampling). In that way, we followed the most recent analytical recommendations to minimize the impact of other factors on vitamin D levels [29]. Our results of no association between vitamin D and HT are in line with several, albeit, smaller studies, the majority of which were not incorporated in the most recent meta-analysis [30,31,32,33].

To take the heterogeneity of the study population into account, we performed analyses using subsets of HT patients according to disease severity. We observed a nominally significant difference in vitamin D levels between MILD and OVERT subgroups of HT cases (Table 2) indicating that there may be subtle differences in vitamin D levels depending on disease severity. Our results suggest that HT patients from the OVERT group that are in advanced stages of disease (overt hypothyroidism and/or treated with LT4 therapy) have lower vitamin D levels (vitamin D median = 19 ng/mL) and a higher proportion of vitamin D deficiency (56.16%) than those in the MILD group (vitamin D median = 20.7 ng/mL; vitamin D deficiency (47.92%)) that are at the beginning of disease with preserved thyroid function (euthyroid/subclinical hypothyroid HT patients). Our results also suggest that it is vital to differentiate HT patients according to the stages of disease when performing analyses, as these subpopulations of patients have a different underlying physiology.

The main criteria for the differentiation of our patients into the OVERT group were TSH > 10 mIU/L or the need for LT4 therapy intake, both of which reflect the malfunction of the thyroid gland. Although the two subgroups of patients have similar median TSH and thyroid hormone values (Table 1), the thyroid gland of patients from the MILD group was still capable of producing sufficient levels of thyroid hormones, whereas the thyroid gland of patients from the OVERT group was no longer functioning adequately and euthyroidism was restored due to synthetic hormone intake (LT4 therapy). There are other clinical features (Table 1) that point out that patients from the OVERT group, in comparison to patients from the MILD group, are in a progressed and more severe stage of HT. They are older and have higher median values for BMI, thyroid antibodies and the number of hypothyroidism symptoms. Taken altogether, these groups consist of two different entities of patients, which reflect the stage (severity) of HT.

The potential downside of our study is that our controls were recruited from December till June (winter–spring) which is the time of the year when vitamin D levels are low, which subsequently means that we do not have data for controls in the other half of the year, including the vitamin D peak in August. Although we have adjusted our analyses for seasonality, and additionally performed a subanalysis using HT cases that were tightly matched to controls on the date of recruitment (winter–spring), an interesting question may arise, and that is how fast vitamin D levels are restored in HT patients in comparison to controls during the sunny part of the year? It may be possible that HT patients in progressed stages of disease (OVERT) may not restore vitamin D values as efficiently as controls or patients at the beginning of disease. The rationale for this statement lies in our observation that controls and HT patients at the beginning of the disease (MILD) have higher vitamin D values, and lower proportions of vitamin D deficiency, than patients in more severe stages of disease (OVERT). Additionally, OVERT HT patients have the highest overall proportion of vitamin D deficiency among all the investigated groups of participants. We believe that future investigations should aim to investigate differences in vitamin D levels between a large cohort of HT patients in advanced stages of disease and healthy individuals, with vitamin D measures equally distributed throughout all the year, to estimate if lower vitamin D values are associated with progressed HT.

We also observed that vitamin D deficiency is high and common for HT patients and control participants, especially in the winter–spring season, which may mask potential significant differences in proportions of vitamin D deficiency between groups: all HT cases (64.69%), MILD (60.64%), OVERT (68.7%) and controls (60.79%). This is in line with Ferrari et al. who showed that 60–90% of the European population have deficient or insufficient vitamin D levels during “vitamin D winter” (November–March) [39]. We see that HT patients from the MILD group have similar levels of vitamin D deficiency as control individuals, whereas the highest proportion of vitamin D deficiency is in the OVERT group. Although our participants live in a region with many sunny days per year, it seems that other factors play a greater role in defining vitamin D levels, probably the most important one being indoor or office work. During the vitamin D peak season (summer–autumn) the proportion of vitamin D deficiency is considerably lower in all HT cases (18.94%), MILD (12%) and OVERT (27.23%). This study recapitulates once more that there is a growing need of promoting outdoor activities for health purposes and/or the need for the intake of vitamin D supplementation. Although, we do not have information on daily sunlight exposure or outdoor activities of our participants, it is important to state that the region of Split, where our participants come from, has a Mediterranean climate with annual sunshine of more than 2600 h or the equivalent of 108 days. Our results, just like the results of Katrinaki et al. who also observed high vitamin D deficiency in another Mediterranean cohort, opens a question of redefining reference vitamin D values [45].

The next important observation of our study is a nominally significant weak negative correlation between vitamin D levels and TSH in all HT patients (Table 4). The majority of studies observed significant associations between vitamin D deficiency and TSH values in HT patients or weak negative correlations between vitamin D levels and TSH [28,46,47,48]. The most similar study to ours, by sample size and result, was reported by Kim et al. [47] who observed a significant negative correlation between vitamin D and serum TSH in the group of 369 AITD patients and 407 controls after adjustment for sample season, age, sex, and BMI (r = −0.127, *p* = 0.013). Chao et al. [28] also observed a negative correlation and a similar effect to ours between vitamin D and TSH in 5230 participants, of which 373 were HT cases.

Similar results of a negative correlation between vitamin D and TSH were found in healthy populations as well [49,50,51,52]. However, one of the biggest studies, a cross-sectional analysis of 8042 individuals, did not observe a correlation between vitamin D and TSH, adjusting for age, sex and seasonal variation [45]. Taken altogether, the majority of literature records on this topic, including our study, suggest that HT patients perform physiologically similarly to healthy individuals in that they have a weak increase of TSH production associated with a decrease of vitamin D. A replication of our results in a greater sample of HT patients is needed for reaching a definite conclusion.

The second, nominally significant, weak positive correlation we observed was between vitamin D and systolic blood pressure in OVERT HT patients (Table 4). Most of the studies that investigated the influence of vitamin D on blood pressure in healthy individuals found an opposite effect, where higher vitamin D levels are correlated with lower blood pressure [53,54]. However, vitamin D deficiency has been associated with hypertension [54], and importantly, our OVERT group has a very high proportion of vitamin D deficiency, which could provide an explanation for our finding. The observed positive correlation is weak and the median systolic blood pressure in our OVERT group is not increased (120 mmHg), therefore, it is not clear if the observed correlation in HT patients with overt hypothyreosis has clinical relevance.

A limitation of our study is that our control group is of a modest size, however the advantage is that all our controls are clinically confirmed HT-free individuals. Another limitation is that the observed correlations are weak and nominally significant. For that reason, our results need to be taken with precaution and replicated in a bigger sample. However, the advantage of our study is that we analyzed vitamin D with the most comprehensive list of clinical phenotypes in one of the biggest cohorts of HT patients to date.

## 5. Conclusions

In conclusion, we have not found an association between vitamin D levels and HT patients of all disease stages; however, results of our study indicate that there may be a subtle decrease in vitamin D levels associated with disease severity. We also observed high proportions of vitamin D deficiency in all study participants, alerting of necessity for outdoor activities for health purposes and/or the need for intake of vitamin D supplementation, especially for HT patients with a more severe form of disease. Our study shows that there is a need for a more detailed categorization of patients according to disease severity when performing scientific research.

## Figures and Tables

**Table 1:** Clinical characteristics of HT patients and control participants.

	HT Patients			
Phenotype	ALL	MILD	OVERT	Controls
	(N = 461)	(N = 240)	(N = 219)	(N = 176)
	Median (Q1–Q3)	Median (Q1–Q3)	Median (Q1–Q3)	Median (Q1–Q3)
Age, years	38.02 (27.76–48.49)	35.81 (25.78–46.95)	40.28 (31.04–50.37)	35.17 (30.12–44.31)
BMI, kg/m^2^	23.52 (20.76–26.85)	23.15 (20.72–26.59)	24.01 (21.02–26.99)	22.66 (20.96–25.24)
TSH, mIU/L	3.33 (1.74–5.68)	3.23 (1.82–4.74)	3.52 (1.67–12.30)	1.48 (1.14–1.95)
T3, nmol/L	1.60 (1.30–1.80)	1.70 (1.50–1.90)	1.50 (1.20–1.80)	1.50 (1.40–1.70)
T4, nmol/L	105 (89–118)	106 (91–117.25)	103 (84.85–121)	101 (89.37–116)
fT4, pmol/L	12.10 (10.20–13.20)	12.10 (10.90–13.10)	11.90 (9.90–13.70)	12.70 (11.90–13.70)
TgAb, IU/ml	135 (36.40–422.40)	121.50 (26.40–321.30)	192 (49.30–596.05)	10.75 (9.10–18.00)
TPOAb, IU/ml	212 (27.60–652.90)	161.50 (17.40–529.75)	273 (66.40–945.50)	3.40 (1.20–8.70)
Thyroid volume, cm^3^	9.85 (7.30–13.91)	9.89 (7.72–13.26)	9.59 (6.82–14.90)	8.69 (6.63–10.60)
No. of symptoms	4 (1–7)	3 (1–6)	5 (2–8)	/
Systolic bp, mmHg	120 (110–130)	115 (110–130)	120 (110–130)	110 (100–120)
Diastolic bp, mmHg	70 (65–80)	70 (65–78.75)	70 (65–80)	65 (60–80)

Q1-first quartile, Q3-third quartile; two HT patients were not reallocated in the MILD/OVERT group.

**Table 2:** Results of logistic regression for vitamin D levels between tested groups.

Tested Group	N 1	N 2	Median 1 (Q1–Q3)	Median 2 (Q1–Q3)	OR (95% CI)	*p*-Value
HT vs. controls ^a^	461	175 ^c^	19.7 (14.4–25.2)	17.3 (13.2–22.7)	0.987 (0.958–1.017)	0.401
HT vs. controls ^b^	303	175	17.1 (13.2–22.2)	17.3 (13.2–22.7)	0.983 (0.954–1.014)	0.277
MILD vs. controls ^a^	240	175	20.7 (14.9–25.8)	17.3 (13.2–22.7)	1.005 (0.970–1.041)	0.788
MILD vs. controls ^b^	155	175	17.7 (14.0–22.6)	17.3 (13.2–22.7)	1.002 (0.966–1.038)	0.927
OVERT vs. controls ^a^	219	175	19 (13.9–24.5)	17.3 (13.2–22.7)	0.971 (0.937–1.006)	0.105
OVERT vs. controls ^b^	147	175	16.7 (12.1–21.7)	17.3 (13.2–22.7)	0.966 (0.930–1.002)	0.065
MILD vs. OVERT ^a^	240	219	20.7 (14.9–25.8)	19 (13.9–24.5)	1.038 (1.005–1.071)	0.023

^a^ adjusted for age, sex, bmi, smoking status and seasonality; ^b^ matched for the season of blood draw (winter–spring) and adjusted as for ^a^; ^c^ data for seasonality is missing in one control sample; *p* < 0.05 are shown in bold, Q1: first quartile, Q3: third quartile, OR (95% CI)—odds ratio with 95% confidence intervals.

**Table 3:** Comparison of proportions of individuals with vitamin D deficiency.

Tested Group	N 1	N 2	Proportion 1 (%)	Proportion 2 (%)	*p*-Value (χ2-Test)
HT vs. controls ^a^	196	107	64.69	60.79	0.394
MILD vs. controls ^a^	94	107	60.64	60.79	0.977
OVERT vs. controls ^a^	101	107	68.7	60.79	0.139
MILD vs. OVERT ^b^	115	123	47.92	56.16	0.077

^a^ matched for the season of blood draw of 6 months (winter-spring); ^b^ HT patients with vitamin D values for all the year.

**Table 4:** Correlation analyses between vitamin D levels and thyroid-related clinical phenotypes.

Phenotype		ALL (N = 461)	MILD (N = 240)	OVERT (N = 219)
TSH	r	−0.113	−0.092	−0.015
	*p*	**0.029**	0.2	0.837
T3	r	−0.008	−0.013	−0.135
	*p*	0.882	0.857	0.07
T4	r	0.026	−0.025	0.053
	*p*	0.622	0.73	0.478
fT4	r	0.011	−0.009	−0.006
	*p*	0.832	0.906	0.941
TgAb	r	−0.048	−0.002	−0.026
	*p*	0.357	0.979	0.724
TPOAb	r	−0.039	−0.058	0.047
	*p*	0.45	0.419	0.53
Thyroid volume	r	−0.004	0.009	0.013
	*p*	0.947	0.899	0.867
No. of hypothyroid sym.	r	0.018	0.11	−0.032
	*p*	0.755	0.178	0.693
Systolic blood pressure	r	0.073	−0.03	0.205
	*p*	0.249	0.731	**0.025**
Diastolic blood pressure	r	−0.001	−0.04	0.112
	*p*	0.987	0.644	0.227

r-Spearman correlation coefficient; nominally significant correlations are shown in bold.

## Data Availability

The data that support the findings of this study are available on reasonable request from the corresponding author.
